# Multifunctional Engineering of Exosomes for Precision Therapeutics: Strategies for Targeted Delivery, Barrier Evasion, and Clinical Translation

**DOI:** 10.1007/s11095-025-03961-w

**Published:** 2025-11-05

**Authors:** Shea Riona Mendonca, Pragathi Devanand Bangera, Mahesha Keerikkadu, Vamshi Krishna Tippavajhala, Mahalaxmi Rathnanand

**Affiliations:** Department of Pharmaceutics, Manipal College of Pharmaceutical Sciences, Manipal Academy of Higher Education, Manipal, 576104 Karnataka India

**Keywords:** biomolecule, cancer, exosomes, gene therapy, immune system

## Abstract

Exosomes (EXM), cell-secreted nanoscale vesicles, are now used as promising tools for therapeutic protein, nucleic acid, and small molecule delivery. However, various challenges, such as rapid immune system clearance, ineffective cargo loading, and reduced targeting specificity, hold them back from being clinically translated. Recent breakthroughs in EXM engineering have made them excellent biomolecule delivery tools. This review critically explores state-of-the-art strategies to maximize cargo incorporation, reengineer EXM surfaces, and create synthetic EXM mimetics. We present important engineering methods, such as genetic manipulation to increase cargo encapsulation, functionalization with targeting ligands, and designing synthetic vesicle structures. We further discuss the therapeutic uses of engineered EXM for different applications, such as cancer treatment, gene therapy, and regenerative medicine, highlighting their potential to evade biological barriers like the blood–brain barrier. Challenges in manufacturing, quality control, and regulatory concerns of translating engineered EXM into clinical therapies are also discussed. We emphasized the upcoming trends that would facilitate improving EXM-based delivery platforms, such as the creation of multifunctional engineered EXM and the incorporation of artificial intelligence for tailored drug delivery. This review stresses the revolutionary value of EXM engineering in establishing next-generation targeted therapeutics, unveiling new fronts for precision medicine and personalized health.

## Introduction

Extracellular vesicles (EVs) represent lipid-enclosed particles released by cells into the extracellular environment, playing crucial roles in intercellular communication. Among EVs, three main subtypes, microvesicles (MV), Exosomes (EXM), and apoptotic bodies, can be distinguished based on their formation processes, release mechanisms, dimensions, composition, and specific functions [1]. Eukaryotic cells secrete heterogeneous membrane vesicles in normal and pathological conditions. The secreted materials consist of various MV, macromolecular complexes, and small molecules that enter the extracellular space continuously. Of these secreted components, EXM is a unique class of nanoscale particles with special characteristics [2]. Based on their size, these membrane-derived vesicles are divided into two broad categories that is MVs, which are larger-sized vesicles (100–1000 nm), and EXM is the smaller type (10–100 nm) [3]. EXM, synonymously referred to as macrovesicles (MAV), are EVs (50–2000 nm) formed by the direct budding of the cytoplasmic membrane to allow intercellular communication [4]. EXM are small membrane-bound vesicles secreted from cells, transporting bioactive compounds such as proteins and nucleic acids. They are constitutively secreted during normal cell function or as a response to stimulation or stress, and are implicated in intercellular communication and possibly in disease mechanisms [5]. They are extracellular vesicles that occur when the plasma membrane and the multivesicular body (MVB) merge, extruding intraluminal vesicles (ILVs) into the extracellular medium. These vesicles transport different biomolecules such as proteins, lipids, and RNA, allowing for intercellular communication and the regulation of cellular processes [6]. The development of EXM is complex in terms of cellular machinery. It seems to be the product of different internal vesicle fusion events, including the interactions among lysosomes, early and late endosomes, and other cell structures, depending on the type of cell [5]. Inside cells, EXM is generated by the inward budding of the endosomal membrane, producing ILVs that contain lipids and proteins. When MVB fuses with the plasma membrane, the ILVs are secreted as EXM into the extracellular medium [7]. EXM are very heterogeneous, likely to express the phenotypic state of the cell that secretes them. EXM are made of a lipid bilayer and can include known components of a cell, such as proteins, RNA, and DNA, at any given time [8].

The classic concept of EXM started with Johnstone's description of their secretion by reticulocytes, with sheep reticulocyte maturation being the first reported instance of EXM generation by inward plasma membrane budding, producing intracellular endosomes that play a role in cell surface protein turnover [8]. EXM is secreted by hematopoietic cells like reticulocytes, B lymphocytes, T cells, and platelets. Non-hematopoietic cells like intestinal epithelial cells, Schwann cells, astrocytes, neurons, melanocytes, mesothelioma cells, adipocytes, fibroblasts, and tumor cells may also secrete EXM [3]. Most cell types have been shown to secrete EXM. Reticulocytes, B lymphocytes, T cells, and platelets are recognized EXM-secretors from the hematopoietic family of cells [5]. The Discovery that EXM contains RNA (both messenger and microRNA) indicates a possible genetic transfer of data exchanged between cells [9]. EXM is secreted by all types of cells in culture and found abundantly in body fluids, including blood, saliva, urine, breast milk, bronchoalveolar lavage fluid, amniotic fluid, epididymal fluid, ascites, and synovial fluid. They can also be found in the peripheral blood because they spill into the bloodstream [5].

This review emphasizes the revolutionary potential of EXM engineering in developing next-generation targeted therapeutics, leading to precision healthcare and personalized medicine.

### Composition and Structure

**Fig.1:**
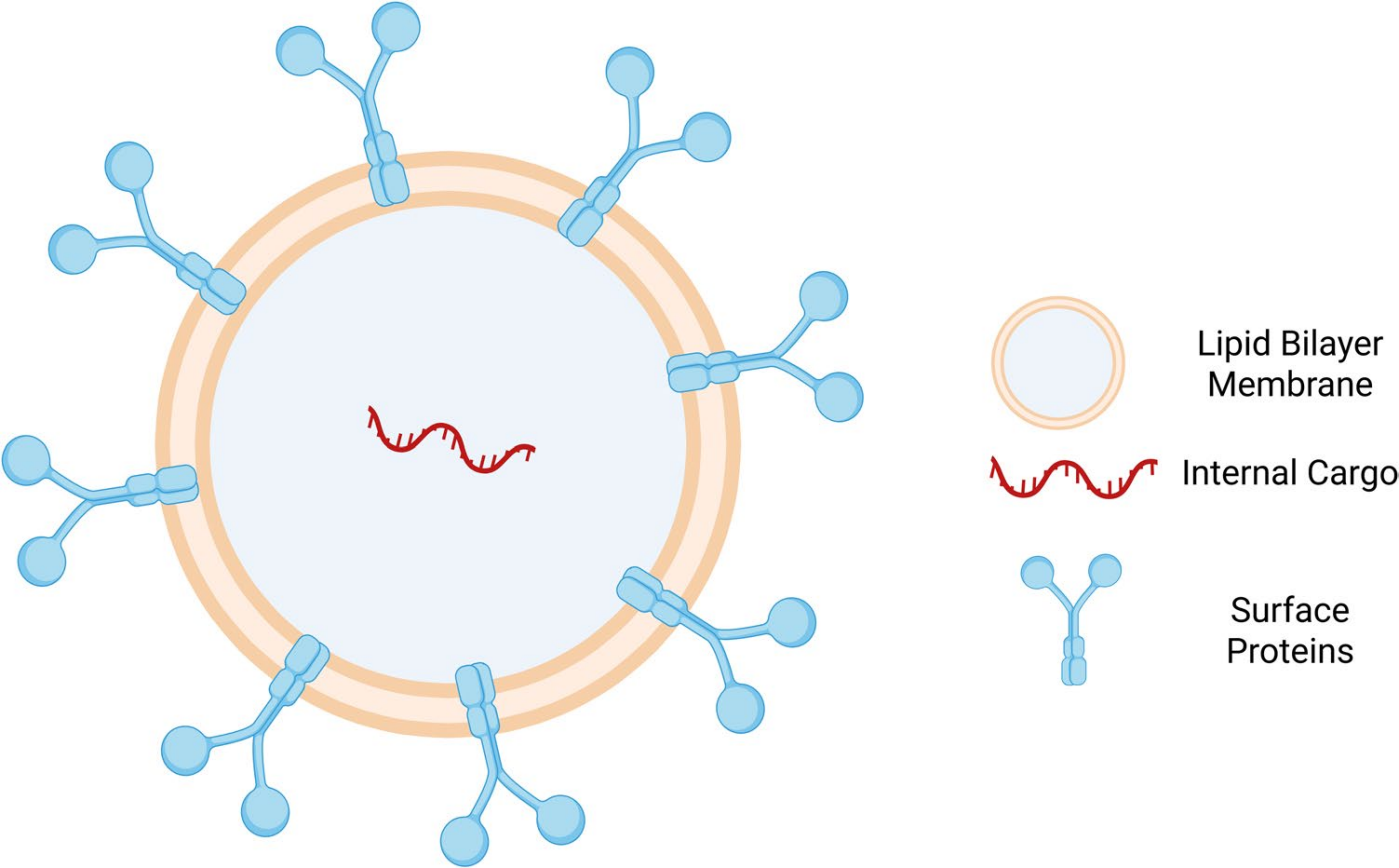
Comprehensive representation of exosome composition and structure. *Representation of the characteristic structure and composition of EXMs, their lipid bilayer membrane, surface-embedded proteins, and biomolecular cargo in the form of proteins, RNAs, and therapeutic entities.

Usually, in extracellular vesicles, size is used to distinguish between the different types of vesicles; apoptotic bodies have a diameter of 50–500 nm, and MVs range from 100 to 1000 nm. The literature has documented EXM diameters ranging from 10 to 140 nm [10]. It is widely acknowledged that EXMs have a diameter of between 50 and 100 nm and are rich in proteins obtained from the plasma membrane or endosomes. The International Society for Extracellular Vesicles has released rules on the nomenclature because specialists have not reached a consensus on it. By examining the vesicle-associated proteins of EXM, particularly those that are known to be either enriched or depleted in EXM, EXM can be described and assessed, offering information about their composition and functional characteristics [10]. EXM biogenesis begins with plasma membrane invagination, forming a cup-shaped structure that incorporates extracellular soluble proteins and cell-surface proteins. This process generates early-sorting endosomes (ESEs), eventually developing into late-sorting endosomes (LSEs). The limiting membrane of these LSEs undergoes inward invagination to create multivesicular bodies (MVBs). These MVBs contain intraluminal vesicles destined to become EXM. MVBs can either merge with the plasma membrane, releasing Intraluminal Vesicles (ILVs) into the extracellular space as EXM, or fuse with lysosomes for degradation [11]. EXM composition is distinct and intricate [12]. Analysis of EXM from various cell types and organisms has revealed an extensive molecular repertoire, including 4,563 proteins, 194 lipids, 1,639 mRNAs, and 764 microRNAs, according to the ExoCarta database (Version 4). This extensive molecular content emphasizes EXM's complexity and its possible implication in many biological processes. ExoCarta is a database of published and unpublished EXM content, with more than 47,000 entries for proteins, mRNAs, and lipids, offering helpful information for EXM characterization [13]. ExoCarta is a published and unpublished EXM content database containing more than 47,000 protein, mRNA, and lipid entries. ExoCarta is valuable information for the characterization of EXM [11]. EXMs are highly organized complex structures with a precisely organized composition of proteins, lipids, nucleic acids, and other substances, selectively sorted according to the cell type and its conditions. As a result of this complex and dynamic constitution, EXM are sometimes viewed as miniaturized versions of the parent cells, bearing the molecular signatures and functional traits of the original cell [14]. The contents of EXM show a regulated sorting process and represent the donor cell composition. Figure 1 represents the elaborate composition and structural profile of EXM, including the lipid bilayer membrane, surface-embedded proteins, and the internal cargo for therapeutic purposes.

### Protein Composition

EXMs have a highly selective and varied protein composition that reflects the identity and physiological state of their cell of origin. EXMs are enriched with proteins from the plasma membrane, cytosol, and endocytic compartments and play an important role in cell communication, signaling, and cargo transport [15]. EXM has distinct protein markers that assist in tracing their biological origin and functional direction. EXMs generated from antigen-presenting cells, such as dendritic cells and macrophages, usually express MHC class II and CD86, which are required for antigen presentation and immunological activation. Immature dendritic cell-derived EXMs include MFG-E8/Lactadherin, a protein implicated in apoptotic cell clearance and immunological regulation [11]. Tetraspanins, such as CD9, CD63, and CD81, were first found in B lymphocytes but are now regarded as universal exosomal markers due to their constant expression across different cell types and involvement in membrane organization, cell adhesion, and signal transduction [16]. Furthermore, structural and trafficking-related proteins, including annexins, Rab family GTPases, flotillin, syntenin, and moesin, are often seen across distinct exosomal populations, enabling vesicle formation and cytoskeletal interaction [17]. From a disease standpoint, several exosomal proteins are preferentially packed under pathological circumstances and act as biomarkers or effectors in disease development. EXM generated from stressed or sick cells upregulates proteins such as thioredoxin peroxidase II, Alix, 14–3-3, and galectin-3, which are typically linked to apoptotic signaling pathways [15]. Heat shock proteins, such as Hsp70 and Hsp90, are also seen in EXMs under cellular stress or malignancy. These chaperones help peptides load onto MHC class I and II molecules, indicating a dual involvement in stress response and immunological regulation [13].

Exosomal proteins have important roles in a variety of cellular activities, particularly signal transduction and membrane trafficking. Rab GTPases, ESCRT components (Alix, TSG101), and annexins all play important roles in MVB synthesis, membrane fusion, and vesicle sorting [13]. Signal transduction. G-proteins, protein kinases, and tetraspanins enhance communication between donor and recipient cells by influencing activities such as proliferation, migration, and immunological responses [16]. EXM docking and uptake by target cells involves adhesion molecules such as ICAM-1, CD146, CD18, CD11a/b/c, CD166, and LFA-3/CD58 [17]. Furthermore, EXMs contain lipid metabolism-related proteins, phospholipases, and lysobisphosphatidic acid (LBPA)-binding proteins, such as Alix, that facilitate membrane curvature and lipid remodeling during vesicle formation [13]. Importantly, EXM is absent of nuclear, mitochondrial, and endoplasmic reticulum proteins, emphasizing its significance in extracellular signaling rather than intracellular metabolism. Instead, they are abundant in cytosolic proteins like as actin, EEF1A1, EEF2, syntenin, moesin, and albumin, which represent both the selective loading processes of exosomal packing and their multifunctionality in intercellular communication [17].

### Lipid Composition

EXM has a unique lipid composition that is both cell type-specific and evolutionarily conserved, mirroring the lipidomic profile of their parent cells. These lipids are important not only for the structural integrity of EXMs but also for their involvement in intercellular communication and the control of lipid homeostasis in recipient cells [11]. EXMs have a nanospherical, bilayer membrane shape with asymmetrical lipid distribution sphingomyelin (SM) is abundant in the outer leaflet, whilst phosphatidylserine (PS) is frequently located in the inner leaflet EXMs include a variety of lipids, including cholesterol (CHOL), diglycerides, sphingolipids (e.g., SM, ceramide), phospholipids, and glycerophospholipids including phosphatidylcholine (PC), phosphatidylethanolamine (PE), phosphatidylinositol (PI), and polyglycerophospholipids [18]. Several investigations have demonstrated that EXM lipid content varies greatly depending on the generating cell type. EXMs produced from mast cells and dendritic cells were among the first to be examined for lipid composition, indicating higher quantities of phosphatidylethanolamines, which flip faster across membrane leaflets than those in the parent cell membrane [4]. This quick flipping may enhance EXM flexibility and membrane fusing capabilities. Furthermore, EXMs produced from B-lymphocytes can contain up to three times as much cholesterol as their parent cell membranes. Cholesterol buildup in multivesicular bodies (MVBs) appears to be important for the creation of intraluminal vesicles, giving birth to EXM [13]. Lipidomic investigations on EXMs released by PC3 prostate cancer cells revealed a substantial enrichment in glycosphingolipids, contributing to their structural stability and resistance to breakdown in the extracellular environment [11]. EXM lipid content is widely acknowledged as playing a role in pathophysiological situations. EXMs may transport bioactive lipids like as prostaglandins and leukotrienes, as well as enzymes involved in lipid metabolism, which can influence inflammation and metabolic signaling in target tissues [19]. EXM-mediated transport of cholesterol and sphingomyelin to recipient cells can affect lipid homeostasis, which is particularly significant in cholesterol storage diseases such as atherosclerosis [13]. Exosomal lipids play an important role in signaling cascades, membrane dynamics, and target cell regulation. High levels of cholesterol and sphingomyelin in EXM membranes aid in the creation of lipid rafts, which are important in signal transduction and membrane fusion. The formation of particular lipid domains, such as the new sphingomyelin-enriched microdomains discovered in EXMs, facilitates specialized activities such as cargo sorting and delivery [13]. Furthermore, lipid enrichment patterns, such as decreased phosphatidylcholine owing to sphingomyelin synthase activity, demonstrate the active modification of membrane composition during EXM biogenesis. Once released, EXMs can carry their lipid payload to target cells, therefore affecting lipid metabolism, membrane fluidity, and cellular signaling networks, which may then impact cell fate decisions and inflammatory responses [14].

### Nucleic Acid

The discovery of nucleic acids, like as mRNAs and miRNAs, in EXM has greatly aided our knowledge of their role as intercellular genetic communication agents. Although EXMs primarily contain small or degraded RNA fragments typically under 200 nucleotides in length, they are capable of transferring functionally active, full-length mRNAs and regulatory RNAs to recipient cells, where they can influence gene expression and protein synthesis via endocytosis-mediated delivery [12]. EXMs, in addition to mRNAs, transport a range of RNA species such as microRNAs (miRNAs), long non-coding RNAs (lncRNAs), transfer RNAs (tRNAs), and even virus RNAs, all of which can contribute to changes in cellular phenotype in the recipient cell [13]. EXM's RNA payload is directly related to the origin of the secreting cell type. EXM are generated by a wide spectrum of healthy and sick cells, including epithelial cells, adipocytes, fibroblasts, Schwann cells, astrocytes, and neurons, as well as hematopoietic cells, including reticulocytes, B and T lymphocytes, platelets, mast cells, dendritic cells, and macrophages [8]. The functional profile and RNA content of these vesicles are heavily influenced by their parent cell. For example, EXM released by B cells contains MHC class I and II molecules as well as T cell-stimulating proteins, which encourage T cell proliferation. In contrast, EXMs produced from carcinoma cells are characterised by chemicals that govern cell adhesion and facilitate tumor development and metastasis [14]. Notably, EXM produced from mesenchymal stem cells (MSCs) has sparked attention for its potential therapeutic uses in cell-free therapies for illnesses such as myocardial infarction, drug addiction, and epilepsy, demonstrating the impact of cellular origin on RNA-mediated bioactivity [13].

In illness settings, EXM-associated RNAs, particularly miRNAs, are emerging as biomarkers and functional drivers in a variety of pathological processes. High-throughput RNA sequencing revealed that miRNAs are the most prevalent RNA species in human plasma-derived EXMs, accounting for 42.32% of total raw reads and more than 76% of mappable reads [13]. These short non-coding RNAs (17–21 nucleotides) use RNA interference to control gene expression after transcription. Exosomal miRNAs, including miR-214, miR-29a, miR-1, miR-126, and miR-320, have been linked to angiogenesis, hematopoiesis, exocytosis, and cancer development via EXM-mediated transport. These miRNAs may move unidirectionally across cells, producing a molecular communication network that causes phenotypic changes in the recipient cells. Interestingly, investigations in 2011 found that a considerable fraction of extracellular miRNAs are not necessarily encased within EXMs and may instead be connected with Argonaute (Ago) proteins, which stabilize miRNAs and improve their targeting efficacy across hundreds of mRNAs [10]. EXM-derived RNAs participate in several signaling cascades and gene regulation mechanisms. The presence of mRNA in EXM allows new proteins to be translated in recipient cells, resulting in horizontal gene transfer. MiRNAs, depending on their sequence and environment, can contribute to gene silencing, inflammatory control, tumor suppression or promotion, and angiogenic signaling. These functions establish EXMs as key vehicles for epigenetic control and cell destiny modification, particularly in the presence of stress, injury, or disease. Advanced characterization approaches, including trypsin digestion, mass spectrometry, Western blotting, and fluorescence-activated cell sorting (FACS), have permitted thorough profiling of EXM contents, showing the selective integration of RNA and protein species into exosomal cargo [20].

### Biogenesis and Release Mechanisms of EXM

Early endosomes develop into MVBs as they mature. These MVBs are transported to the trans-Golgi network for endosome recycling and then delivered to lysosomes for the degradation of carried materials [21]. MVBs employ various mechanisms to produce ILVs through inward budding [22]. Wolf first observed extracellular vesicles in plasma 50 years ago, referring to them as platelet dust [23]. EXM biogenesis involves three main processes: formation within endocytic vesicles, generation of multivesicular bodies, and release of EXM [24]. Alternatively, MVBs can be degraded through fusion with lysosomes. ILV formation pathways can be categorized into two types: ESCRT-dependent and ESCRT-independent. The ESCRT-dependent pathway includes both ubiquitin-dependent and ubiquitin-independent mechanisms. Studies have shown that ILVs can form even when all major ESCRT-associated subunits are eliminated, suggesting the existence of ESCRT-independent processes [22]. EXM formation starts with the inward budding of the plasma membrane to generate early endosomes. These endosomes partially invaginate and bud into surrounding lumina, taking cytoplasmic content to generate ILVs [21]. These intraluminal vesicles transport cargoes and interact with trafficking effectors at endosomal and plasma membrane sites. These interactions cause membrane bending and scission, eventually resulting in EXM formation and release [25].

### Formation of Early Sorting Endosome

EXM biogenesis begins in the endosomal system,Ormation of Early Sorting Endosomestarting with early-sorting endosomes (ESEs), initial sites for sorting membrane-associated cargo and solute transport in mammals. The ESE, which is generated by the fusion of primary endocytic vesicles, is a large vesicle composed of heterogeneous receptors, lipid membranes, extracellular fluid, adhesion molecules, polarity markers, ion channels, nutrient transporters, and cell signaling receptors. Endocytic vesicle formation is carried out by two main pathways: clathrin-mediated endocytosis (CME) and clathrin-independent endocytosis (CIE). The CME pathway, for which a wide range of characterization has been accomplished previously, encompasses cargo selection, clathrin coat assembly, vesicle scission, and uncoating, which is aided by proteins like HSC70 and auxilin. The CIE pathway includes caveolar, CLIC/GEEC, and ARF6-dependent mechanisms, playing a role in endosomal vesicle formation. These vesicles then mature and contribute to EXM formation [22].

### MVB Formation

#### ESCRT-dependent Pathway

Formation of the multivesicular body (MVB) is controlled by the endosomal sorting complex required for transport (ESCRT), which is composed of four soluble multiprotein complexes-ESCRT-0, ESCRT-I, ESCRT-II, and ESCRT-III that sort proteins into ILVs. These complexes are recruited to the cytosolic side of the endosomal membrane and typically require ubiquitination of the cytosolic tail of endocytosed receptors for efficient sorting. Tsg101 from the ESCRT-I complex binds to ubiquitinated cargo proteins, triggering activation of the ESCRT-II complex, subsequently leading to ESCRT-III complex formation [26]. This complex plays a crucial role in sequestering MVB proteins while recruiting a deubiquitinating enzyme to remove the ubiquitin tag before sorting proteins into ILVs. An ATPase eventually disassembles the ESCRT-III complex [1]. Although ESCRT proteins are known to target membrane proteins for lysosomal degradation, their specific role in EXM formation remains somewhat unclear. However, proteomic studies have demonstrated that components of the ESCRT complex, such as Alix and Tsg101, are present in dendritic cell EXM, suggesting that EXM formation depends on the ESCRT pathway [27].

#### ESCRT Independent Pathway

ILV synthesis typically involves ESCRT complexes, and certain proteins and lipids can facilitate this process independently. Tetraspanins, particularly CD63, contribute to EXM biogenesis by directing cargo to multivesicular bodies, organizing the endosomal membrane into functional tetraspanin-enriched domains (TEMs), and promoting the release of specific molecules via EXM. CD63 has attracted significant attention for its role in tumor signaling and vesicular transport. Additionally, Gi-coupled S1P1 receptors have been linked to MVB maturation, though the exact mechanism remains unclear. Ceramides, a type of sphingolipid, play an important role in ESCRT-independent membrane deformation by creating lipid raft microdomains in the plasma membrane [28]. These domains induce spontaneous negative curvature and ILV production without requiring ESCRT-III. The microtubule-associated protein LC3 recruits factors related to neutral sphingomyelinase (nSMase, a ceramide-producing enzyme) to endosomal membranes, promoting ceramide-mediated ILV production. Recent studies have also shown that Rab31 GTPase activation initiates membrane budding in microdomains, and the ceramide transfer protein (CERT) facilitates ceramide transport from Golgi and ER networks to endosomes, a crucial step for EXM formation and secretion [29].

Tetraspanins are also central to an ESCRT-independent mechanism of cargo sorting and EXM formation by interacting with cytosolic and transmembrane signaling proteins. These interactions form tetraspanin-enriched microdomains (TEMs) that act as platforms for cargo transport, ordering membrane microdomains to enable efficient cargo sorting. For instance, the membrane metalloproteinase CD10 enters intraluminal vesicles by binding to CD9, whereas the melanocyte-specific glycoprotein PMEL enters ILVs by binding to CD63. Moreover, tetraspanins like CD9 and CD63 aid in intracellular transport of LMP1. These tetraspanin-structured supramolecular assemblies have a remarkable impact on exosomal cargo trafficking, aiding in EXM development and cargo selection [22].

#### Cargo Setting of EXMs

Cargo recruitment is vital for the process of inward membrane budding to begin in endosomes, and it is regulated by different mechanisms of endosomal sorting because the cargo is highly heterogeneous in composition and includes proteins, lipids, and nucleic acids. Proteins are targeted to the endosomal pathway via monoubiquitination, whereas polyubiquitination targets cargo for degradation; the ubiquitin-interacting-motif (UIM) domain of ESCRT-0 complex protein Vps27/Hrs binds to and accumulates the ubiquitinated cargo. In clathrin-dense microdomains of the endosomes, intraluminal vesicles (ILVs) assemble distant from the cytoplasm when Vps27/Hrs facilitates accumulation and sorting of the ubiquitinated cargo into ILVs for further processing [29]. Proteins are directed to the endosomal pathway through monoubiquitination, while polyubiquitination marks cargo for degradation. The ubiquitin-interacting motif (UIM) domain of the ESCRT-0 complex protein Vps27/Hrs binds to and concentrates ubiquitinated cargo. Within clathrin-rich microdomains of endosomes, intraluminal vesicles form away from the cytoplasm when Vps27/Hrs helps concentrate and sort ubiquitinated cargo into ILVs for further processing. EXM contains various transmembrane proteins with scaffolding functions, including flotillin 1, IL-6R, EGFR, T-cell receptor, chimeric antigen receptor, GPCR receptors, PD-L1, TGFB, and ADAM proteases. They also feature diverse membrane-interacting proteins with glycosylphosphatidylinositol (GPI) anchors, such as glycans, glypican-1, DAF, and MAC-IP, along with small GTPases associated with the inner leaflet of EXM membranes through posttranslational prenylation. Additionally, proteins that undergo N-terminal myristoylation, like BASP-1 and Src signaling kinases, are sorted into EXM, while lentiviral gag proteins incorporate into EXM via N-terminal myristoylation. EXM also contains ESCRT complex-interacting or ESCRT-forming molecular chaperones, including syntenin, TSG101, and ALIX, as well as molecular chaperones like HSP70, HSP90, and HSP20, which are involved in biogenesis and protein sorting within EXM. Cargo sorting involves numerous posttranslational modifications, including ubiquitination, SUMOylation, and phosphorylation [25].

After Cargo sorting and ILV generation, MVBs merge either with lysosomes to degrade cargo or with the plasma membrane to discharge ILVs as EXOs. Recent studies indicate that ILVs can have more than one fate. ILVs can be retrofitted with the MVB membrane or be secreted from cells through lysosomal exocytosis [29]. Post-translational modification in the form of ISGylation has also been found to facilitate the fusion of multivesicular bodies with lysosomes to degrade cargo rather than secrete it. One study recently revealed that MVB positioning is dictated by TSPN6 concentration: high TSPN6 levels favor lysosomal degradation, while low concentrations enable the release of EXM syntenin and SDC4. These results imply that blocking MVB-lysosome fusion might facilitate the export of cellular products through EXM, possibly for better secretion and cargo delivery [22]. After the inward budding of ILVs, transport and fusion with the plasma membrane of secretory MVBs are important for EXM release. The process entails numerous principal components such as the SNARE complex, dynein, kinesin, microtubules, microfilaments, and small GTPases, with the Rab GTPases playing a central part in vesicle traffic, including budding, motility, and fusion. Rab GTPases such as Rab27a, Rab27b, and Rab35 recruit tethering proteins to bind with SNARE proteins, allowing vesicle docking at the plasma membrane. SNARE proteins, such as v-SNAREs (VAMPs) and t-SNAREs (syntaxins and SNAPs), facilitate endosomal and plasma membrane fusion in the secretion of EXM. One v-SNARE typically complexes with three t-SNAREs to facilitate the membrane fusion and subsequent release of EXM. Rab27 controls the transit and fusion of secretory vesicles by interacting with effector proteins such as Slp4, Slac2-b, and Munc13-4. Simultaneously, Rab35 regulates the docking of endocytic vesicles onto the plasma membrane in oligodendroglial cells in a GTP-dependent manner. Directed transport of secretory MVBs comprises movement along the plasma membrane by molecular motors, dependent on the microtubule cytoskeleton polarity and positioning of MVBs in the cell. Rab GTPases and their effectors regulate MVB and plasma membrane fusion, and the SNARE protein family is involved in membrane fusion. Post-translational modifications, including O-GlcNAcylation and phosphorylation of SNARE proteins, control EXM secretion. For instance, decreased O-GlcNAcylation of SNAP-23 increases its interaction with syntaxin 4 and VAMP8, augmenting EXM secretion [22]. When v-SNAREs and tSNAREs combine, the interacting membranes form the SNARE complex. EXM is released into the extracellular environment by MVBs through fusion with the plasma membrane [21].

### Natural Functions of EXMs

#### Role in Intercellular Communication

Initially, EXM was primarily considered a mechanism for cells to eliminate waste, especially in those with limited lysosomal degradation capacity. However, they are now recognized as essential mediators of cell-to-cell communication, influencing various signaling pathways through mechanisms including (i) EXM membrane proteins activating intracellular signaling by interacting with receptors on target cells; (ii) proteases cleaving EXM membrane proteins and releasing soluble fragments that bind to cell surface receptors; and (iii) EXM internalization by target cells, triggering downstream effects. EXM is crucial in transferring membrane material between cells, which is essential for their biological activity. This membrane transfer has been demonstrated in vitro and in systems with or without direct cell–cell contact. CD8 + T cells acquire MHC class-I peptide complexes and other surface molecules from antigen-presenting cells in antigen presentation. The T-cell receptor internalizes these complexes, while other molecules transfer via specialized receptors, making T cells susceptible to lysis by cytotoxic T lymphocytes targeting the same MHC-peptide complexes. This process can help eliminate CTLs and reduce immunological responses [30]. EXM is secreted by various cell types, including hematopoietic, epithelial, neuronal, cancer, and stem cells, and is involved in physiological and pathological processes. EXM plays a crucial role in modulating immune responses in the immune system, impacting both adaptive and innate immunity. When pathogen-infected cells release EXM, they carry pathogenic antigens that can be presented to T-cells via MHC molecules once captured by dendritic cells (DCs). Similarly, EXM released by tumor cells carries tumor antigens, which are captured by DCs and displayed, leading to enhanced antitumor immune responses [27].

#### Molecular Transport

EXM functions as bioactive vesicles transporting various molecular cargoes, with proteins representing a significant component (57). The composition of exosomal cargo can reveal homeostatic imbalances across different bodily systems. There are three main methods of loading therapeutic cargo into EXMs: (A) post-isolation loading with intact EXM harvested from parental cells; (B) pre-loading parental cells with the therapeutic prior to EXM secretion and encapsulation; and (C) directly genetically modifying the parental cells with DNA or other methods expressing the therapeutic molecules for incorporation into EXM that is released. Each approach offers distinct advantages and disadvantages, depending on the therapeutic cargo type, disease site, and conditions required for EXM encapsulation [31]. EXM can transport proteins, DNA, various RNA species (including noncoding RNAs), and lipid molecules. They contain both cytosolic and membrane-bound proteins, as demonstrated in numerous studies. Dendritic cell EXM, for instance, features proteins such as hsc73, a member of the hsp70 family, which is important for vesicle formation. Additionally, the exosomal membrane of dendritic cells contains tetraspanins like CD9, CD63, CD81, and CD82, which are critical for immune function and play significant roles in cell communication and response regulation [16]. Several strategies have been proposed for loading naïve EXM in vitro with therapeutic drugs. Lipophilic small compounds, such as curcumin, doxorubicin, paclitaxel, and the model drug Rhodamine 123, are often passively loaded into EXM or EXM-like vesicles through co-incubation at room temperature. Drug loading efficiency varies from 7.2% for paclitaxel to 11.7% for doxorubicin, as measured using HPLC. Although EXM represents unique nanocarriers that naturally transport various proteins and nucleic acids, their relatively limited drug loading capacity may result from their inherent cargo composition and membrane characteristics [31]. Cancer cells utilize EXM to transport oncoproteins to neighbouring cells, promoting neoplastic transformation. EXM also demonstrates immunological activity, capable of presenting antigens. A study by Skotland *et al*. identified 107 lipid species in urinary EXM from prostate cancer patients, including cholesterol, phospholipids, and sphingomyelin, highlighting their potential applications in cancer diagnostics and therapeutics [16]. EXM naturally transports diverse nucleic acids, including mRNA, miRNA, noncoding RNA, mitochondrial DNA, and genomic DNA, making them promising carriers for nucleic acid delivery. Alvarez-Erviti *et al*. pioneered electroporation technology for loading siRNA into dendritic cell-derived EXM, and similar methods have been used to incorporate miRNA into EXM for targeted delivery to breast cancer cells expressing EGFR [31]. Breast cancer EXM from estrogen receptor-positive (ER +) cell lines were found to contain 27-hydroxycholesterol (27-OHC), which regulates p53 expression and promotes the growth of ER + breast cancer cells. Oncogenic cells release miRNAs through EXM that influence tumor suppressor genes in neighboring cells, aiding cancer progression, and most exosomal miRNAs are used as diagnostic markers, with exosomal mRNA content differing from that of the mother cell. However, their miRNA composition remains consistent [16].

### Techniques for EXM Isolation

#### Ultracentrifugation

Ultracentrifugation is the gold standard for separating EXM and is used in 60% of EXM processing. It effectively isolates EXM from various biological materials [32]. Johnstone first used this method to isolate EXM from reticulocyte culture medium in 1987. Théry *et al*. later optimized it in 2006 by increasing centrifugal forces to separate different extracellular components based on density, size, and shape (48). First, the samples were centrifuged at 300, 2000, and 10,000 g, respectively [33]. This procedure involves using 500 × g to isolate cell debris, 10,000 × g to purify the matrix, and 100,000 g to purify the EXM [34]. Ultracentrifugation can be classified into density gradient and differential centrifugation [35]. Differential UC is the first and most described exosomal isolation technique. The classic UC method uses sedimentation to separate EXM from various biological materials, including blood serum, plasma, breast milk, cerebrospinal fluid, amniotic fluid, urine, aqueous humor, and cell culture lines [32]. Analytical ultracentrifugation analyzes particulate materials and polymeric interactions, while preparative ultracentrifugation separates biological components like viruses, bacteria, subcellular organelles, and extracellular vesicles [36].

**Fig. 2:**
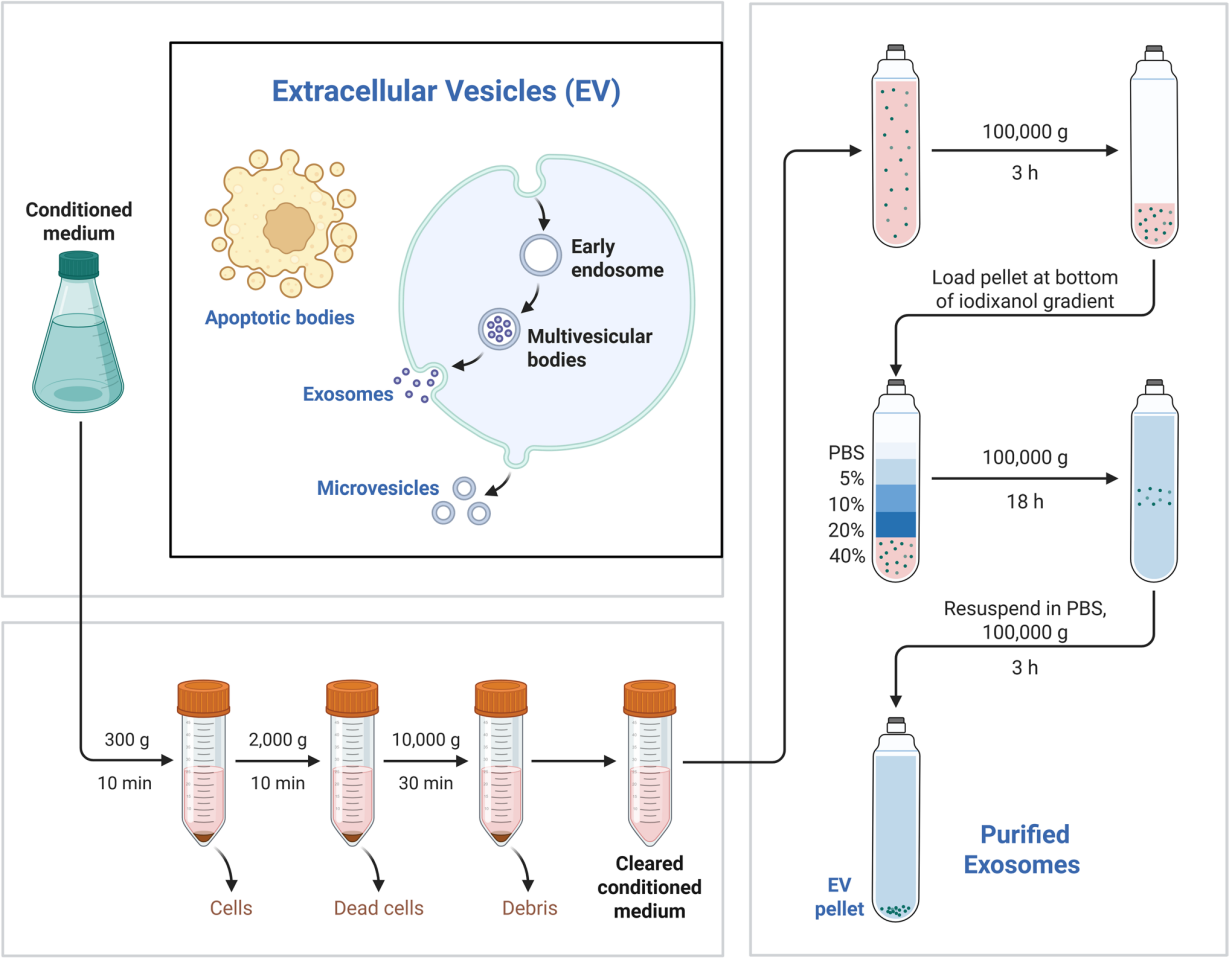
Schematic representation of the differential ultracentrifugation method for exosome isolation. *This procedure entails successive centrifugation steps at higher velocities to get rid of cells, debris, and large vesicles from biological fluids like plasma, serum, or culture media. Low-speed centrifugation (300–2,000 × g) is usually employed to remove cells and debris, followed by medium-speed spins (10,000–20,000 × g) to eliminate microvesicles. Lastly, ultracentrifugation at 100,000 × g or more is utilized to pellet EXM. The final EXM pellet can be washed and re-centrifuged for increased purity.

Differential centrifugation typically involves progressively increasing speeds. This approach uses centrifugation to eliminate cell debris and larger vesicles. Initially, biological fluid samples undergo centrifugation at 500 g, 3000 g, and 16,000 g for up to 1 h to remove cells, debris, apoptotic bodies, and biopolymer aggregates. Finally, EXM are extracted from the supernatant through centrifugation at 100,000–150,000 g for 1–6 h. EXM pellets can be washed and resuspended in sterile filtered phosphate-buffered saline for further analysis or stored at −80°C for future examination [32]. To achieve the desired yield, greater sample quantities are employed at the start of processing to account for the loss of EXM caused by repeated supernatant removal and tube transfers. Despite its limitations, differential centrifugation is a reliable method for producing EXM with reasonable yield and purity [37]. Another related procedure is density gradient centrifugation, which involves inserting a pre-made density gradient (often sucrose or iodixanol) into the centrifuge tube. This approach effectively separates EVs (e.g., EXM) from protein aggregates, non-membranous particles, and biofluids using size and density criteria [34]. This technique isolates particles of different sizes and shapes with equal densities. As samples move through the gradient zone, denser particles pass through the gradient layer and reach the tube bottom faster. Centrifugation separates solutes based on density and mass/size, facilitating separation of vesicles with similar density but different diameters. For optimal EXM isolation, centrifugation should stop when the gap between zones reaches maximum separation, or a high-density medium should be placed at the tube bottom. Alternatively, particles with identical density but different sedimentation coefficients can concentrate in the same zone [35]. Density gradient centrifugation purifies EXM and typically complements ultracentrifugation to enhance EXM purity. There are two primary varieties, with sucrose commonly used as a medium in research applications. However, EXM and retroviruses share similar size and density characteristics, making them difficult to distinguish using sucrose density gradients. Cantin *et al*. discovered that their sedimentation velocities in iodixanol gradients differ significantly, allowing effective separation of EXM from HIV-1-infected cells and yielding high-purity EXM. While density gradient centrifugation improves EXM purity, the high viscosity of sucrose solution reduces sedimentation rates [14]. This approach works well for high-concentration sample analysis due to its simplicity and lack of additional markers [35]. However, it cannot distinguish EXM from viruses or MV because of their similar buoyant densities. Additionally, density-gradient centrifugation requires expensive ultracentrifugation equipment and highly trained technicians [14]. Figure 2 presents the ultracentrifugation-based procedure for the isolation of EXM, which is based on sequential steps of centrifugation to isolate EXM from cells, debris, and larger vesicles.

#### Size Exclusion Chromatography

Size exclusion chromatography (SEC) represents another approach for separating molecules based on their dimensions (71). A mixture passes through a column containing beads with various pore sizes. Molecules travel through polymeric beads according to their size, with smaller particles entering and migrating through the pores, resulting in later elution from the column. EXM with larger hydrodynamic radii travel through the column more quickly due to their inability to enter pores [38]. SEC is a size-based isolation approach with several advantages, including high-purity EXM, gravity-based preservation of biological activity, excellent repeatability, and moderate sample capacity [34]. The technique utilizes the starting biofluid as the mobile phase and a porous gel filtration polymer as the stationary phase. The stationary phase composition allows for differential elution: larger particles elute first, followed by smaller vesicles, and finally non-membrane bound proteins. Larger particles encounter fewer pores to traverse, resulting in a shorter path to the column end and faster elution compared to smaller ones. Various gel polymers including Sephadex, agarose, Biogel P, and allyldextran can be used to prepare the stationary phase or chromatography column [39].

#### Precipitation

Polymer precipitation involves using polyethylene glycol (PEG) as a medium and harvesting EXM using centrifugation to reduce their solubility. This approach was initially used to isolate viruses. Scientists frequently employ this technology to extract and purify EXM due to their similar biophysical features to viruses. Commercial solutions for EXM isolation include the Total EXM Isolation Kit by Thermo Fisher Scientific, Exo-spin by Cell Guidance System, and ExoQuick by System Biosciences [40]. PEG attracts water molecules and precipitates insoluble particles from EXM. The process typically involves overnight co-incubation of materials with 8–12% 6 kDa PEG solution at 4°C. EXM can be isolated using a charged-based precipitation approach in addition to the methods mentioned above. Negatively charged EVs can easily precipitate when interacting with positive compounds like Protamine. EXM recovered using this approach were more efficiently recovered than those extracted using ultracentrifugation (NTA) [38]. The polymer precipitation method is simple to use, requires little analysis time, and can handle large samples [34]. However, precipitated EXM may contain biopolymers, complicating downstream analyses such as mass spectrometry, proteomics, and RNA assays. Adding a pre-filtration step with a 0.22-µm filter or post-precipitation purification (such as centrifugation, filtration, or gel filtration) can reduce contamination from non-exosomal components [41].

#### Ultrafiltration

Ultrafiltration isolation employs nanomembranes or molecular-weight cut-off (MWCO) membranes to capture EXM based on size or molecular mass. This method leverages the fact that EXM, with diameters of 30–100 nm and higher molecular mass than typical proteins, can be isolated using various filter diameters to separate them from other macromolecules in cell culture supernatants or bodily fluids [14]. Ultrafiltration begins by separating EXM from larger contaminants like cells, detritus, and microparticles using membrane filters with pore diameters of 0.1, 0.22, and 0.45 μm. Afterward, EXM are further isolated by applying high centrifugation forces (100,000 to 200,000 xg) to pellet them, with ultrafiltration membranes filtering out EXM by pushing the sample fluid through pores smaller than 100 nm [32]. Even though this method is quicker than ultracentrifugation, it is harmful to EXM because of shear stress and membrane adhesion. This can result in the loss of EXM, a decrease in yield, and increased processing time [37].

#### Affinity Based Separation

Immunoaffinity is a technique that isolates and cleans up biological particles by an antigen–antibody reaction. EXM membranes possess different proteins and receptors, which are common and specialized. Proteins such as tetraspanins and annexins can isolate EXM by binding to specific antibodies [33]. EXM membranes have different proteins and receptors, which are common and specialized. Proteins such as tetraspanins and annexins can isolate EXM by binding on specific antibodies. Several methods for isolating EXM from body fluids include size-based, density-based, and immuno-affinity capture with proteins and antibodies. Surface proteins of EXM, such as CD63, CD81, CD82, CD9, Alix, annexin, EpCAM, and Rab5, mediate high-purity and specific subpopulation isolation. This paradigm for isolation enables antibodies to be immobilized on magnetic beads, chromatography matrices, plates, and microfluidic devices. Magnetic beads are widely used for cell sorting in flow cytometry because of their larger diameter (10–20 µm) compared to EXM (smaller than 1 µm). Magnetic beads offer a larger surface area and a nearly homogeneous capture process and, thus, more efficient and sensitive immune affinity techniques compared to microplate-based methods. In addition, there are fewer volume limitations [36].

#### Microfluidics

**Fig. 3:**
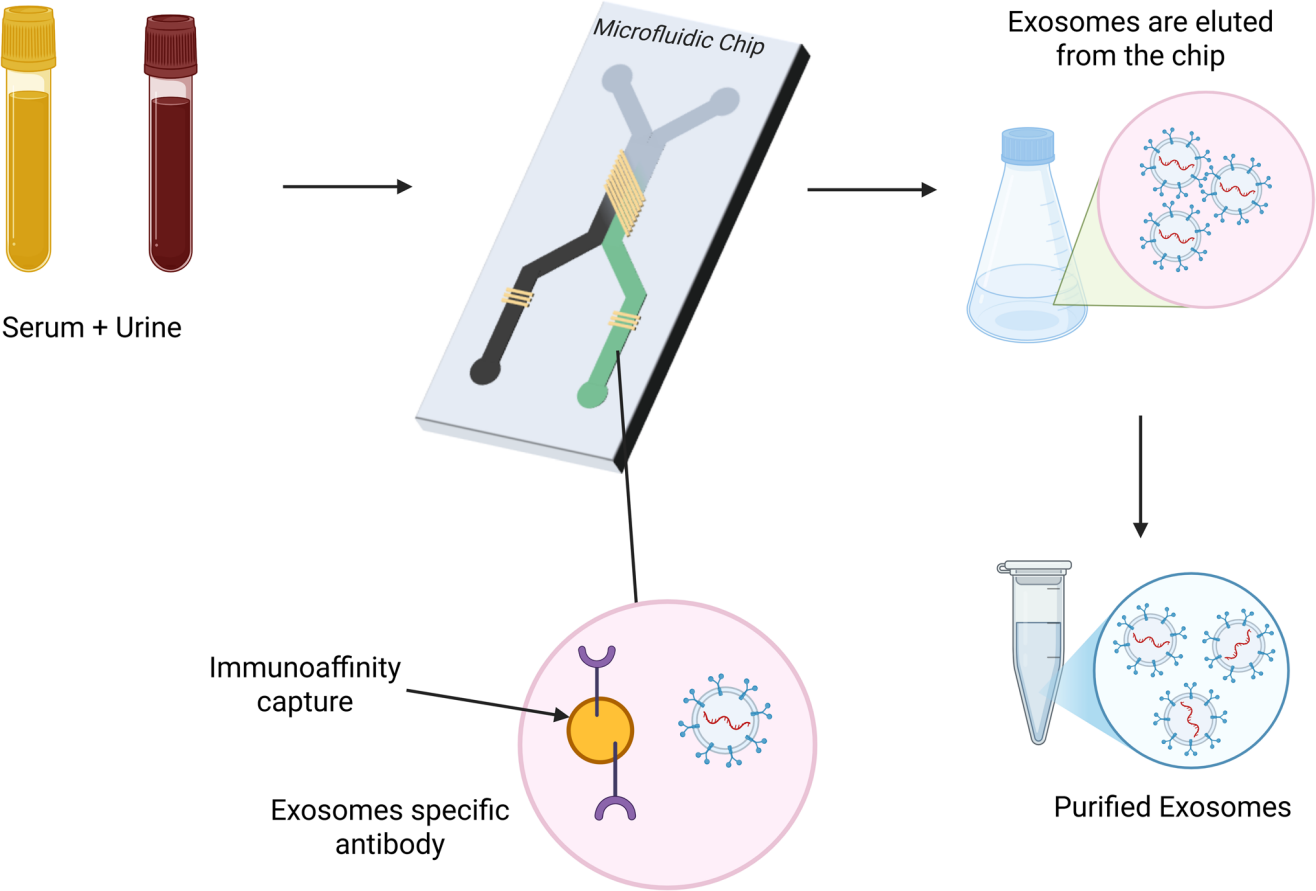
Schematic representation of the immuno-microfluidic method for EXM isolation and detection. *This diagram depicts a microfluidics-based platform that is integrated with immunoaffinity methods for selective binding and analysis of EXM. Microchannels functionalized or magnetic beads covered with antibodies specific to EXM facilitate high-specificity capture of target EXM directly from complicated biofluids like blood, serum, or urine. The captured EXM may then be eluted, quantified, and further analyzed by methods like ELISA, fluorescence tagging, or nucleic acid profiling.

Immuno-microfluidic method fuses microfluidics with immunoaffinity capture to capture EXM using their surface markers, size, and density [39]. The above-mentioned methods of EXM isolation are restricted by low yield, low purity, complex instrumentation, and long processing time. But microfluidic methods provide a future solution to get rid of the above constraints [34]. Microfluidics-based technologies are used extensively today to isolate, detect, and examine EXM on a microlevel. These technologies utilize EXM's physical and biological characteristics [36]. In these systems, EXM is separated by utilizing antibodies immobilized on microfluidic chips that specifically bind to EXM markers. ExoChip with CD63 antibody is a common microfluidic device employed for the isolation of EXM. Microfluidic devices like those comprising gold electrodes or graphene oxide/polydopamine nanointerfaces coupled with antibodies such as CD9 and CD81 provide effective and fast EXM isolation. These techniques offer high purity in the final exosomal preparation, and they have advantages for accurate EXM analysis and applications [39]. Microfluidic technology can be categorized into three types: size-based, immune-affinity-based, and dynamic-based. Its advantages include low reagent volume requirements, multiplex operation capability, reduced production costs, and the potential to integrate various sensors and transducers with different fluidic control mechanisms. Immuno-microfluidic platforms facilitate speedy and selective capture of EXM from intricate biofluids via antibody-coated microchannels. The technique provides high sensitivity, low sample consumption, and higher purity than traditional methods, which is suitable for clinical diagnosis and therapeutic tracking. Figure 3 shows the process flow of EXM capture and analysis. Microfluidic platforms show promise for analyzing EXM in clinical applications by reducing sample consumption, improving data analysis, minimizing cross-contamination, and automating isolation and detection processes [34]. Table I compiles the different methods of isolation used for drug delivery systems based on EXM, along with important physicochemical parameters and their respective therapeutic effects in different disease models.

**Table I.** Summary of Isolation Methods for Exosome-Based Drug Delivery Systems and Their Therapeutic Outcomes

Drug	Diseases	Method of isolation	SIZE, %EE, ZP	Outcome	Ref
Methotrexate	Rheumatoid arthritis	Ultracentrifugation	90–150 nm, −35 to −45 mV, 80%	Enhanced delivery to cancer cells with reduced toxicity	[42]
Hydrocortisone	Addison’s disease	Size exclusion chromatography	120–180 nm, −25 to −35 mV, 85%	Improved anti-inflammatory response in autoimmune diseases	[43]
Curcumin	Ulcerative colitis	Precipitation	100–160 nm, −40 to −50 mV, 70%	Increased bioavailability and therapeutic potential	[44]
Melatonin	Sleep disorders	Ultrafiltration	110–160 nm, −20 to −30 mV, 75%	Enhanced sleep regulation and antioxidant effects	[45]
Rituximab (Monoclonal antibody)	Non-Hodgkin's Lymphoma	Affinity-based separation (anti-CD20)	90–140 nm, −30 to −40 mV, 85%	Improved targeting for autoimmune and cancer therapies	[46]
Insulin	Type-1&Type-2 Diabetes mellitus	Microfluidics	120–180 nm, −30 to −40 mV, 90%	Enhanced cellular uptake and sustained release for diabetes	[47]
Cisplatin	Testicular cancer	Ultracentrifugation	80–120 nm, −40 to −50 mV, 80%	Increased anticancer efficacy with reduced systemic toxicity	[48]
Propranolol	Hypertension	Size exclusion chromatography	90–130 nm, −25 to −35 mV, 75%	Enhanced drug delivery for cardiovascular diseases	[49]

### Strategies for EXM Characterization

#### Nanoparticle Tracking Analysis (NTA)

This biophysical technique evaluates the concentration and size distribution of EXM ranging from 2 µm to 10 nm. Bio applications involve characterizing EVs, viruses, nanoformulations, liposomes, and other drug delivery systems. Particle velocity is measured by tracking exosomal mobility. NTA analyses images of individual EXM using scattered light or emitted fluorescence to determine their Brownian motion and link it with particle size. This approach determines the size, distribution, concentration, and phenotype of EXM. NTA can detect particles as small as 30 nm in a short period [40]. Currently, only two companies (Particle Metrix GmbH and Malvern Instruments Ltd.) provide commercial NTA instruments, which Malvern markets as NTA-based Nanosight devices (LM10, LM20, NS200, NS500) [14]. According to the NanoSight NS300 (Malvern Panalytical) user handbook, each sample should be loaded using a syringe pump with five 60-s movies captured under the dispersed option. A complete sample measurement takes approximately 15 min [50]. This technology appeals to researchers because it allows recovery of materials in their original state following measurements. Furthermore, the approach can detect specific antigens on extracellular vesicles by using fluorescently tagged antibodies. Successful NTA implementation requires accurate sample preparation and appropriate dilution factors [36].

#### Dynamic Light Scattering (DLS)

Also known as photon correlation spectroscopy, this method involves transmitting a monochromatic, coherent laser beam through an EXM suspension [40]. Brownian motion causes particles in a solution to move randomly, causing collisions and energy transfer. This leads to the movement of solute particles. Smaller particles have a greater impact on energy transmission in the sol vent due to their quicker movement [36]. DLS calculates particle radius and size distribution using mathematical algorithms such as the Stokes–Einstein equation. EXM size measurements in DLS can be influenced by two factors: biological heterogeneity and size distribution in the solvent (Because of their faster Brownian motion, smaller particles have a greater influence on energy transmission within the solvent). EXMs vary in size and cargo, resulting in varying light scattering intensities that can be determined through intensity autocorrelation function analysis. DLS particle size measurement requires knowledge of the refractive index (RI) of the particles. Extracellular vesicles exhibit diverse RI values due to size differences and variations in internal contents [51]. The combination of DLS with Bradford assay enables accurate assessment of protein concentration and distribution within vesicles [40]. DLS performs better with monodisperse samples (containing similarly sized particles) compared to polydisperse samples. In heterogeneous EXM preparations with EXM species ranging from 30–100 nm, larger EXM scatter more light than smaller ones, leading to higher average EXM size measurements. Before performing DLS analysis, it’s essential to evaluate EXM preparation quality using complementary techniques like electron microscopy. DLS can determine particle size distribution for particles between 1 nm and 6 μm [51].

#### Transmission Electron Microscopy (TEM)

The nanometer-scale resolution of transmission electron microscopy makes it a popular tool for studying the morphology of EXM. Conventional and cryo-TEM, the two main types of TEM, each offer complementary insights into the structure of EXM [50]. EXMs are fixed, dehydrated, and stained with heavy metals like uranyl acetate in a traditional (negative staining) TEM, providing high contrast images to evaluate size, shape, and structural integrity. This technique is quick (two to three hours), simple to use technically, and appropriate for routine morphological assessment and contamination identification. A common protocol involves treating EXM with 2% paraformaldehyde, mounting on Formvar-carbon-coated grids, followed by glutaraldehyde fixation, air-drying, and staining [36]. Nevertheless, cryo-TEM is becoming more popular because it can image EXMs in a hydrated, vitrified state without fixation or staining while maintaining native morphology. By doing this, sample preparation-related artifacts are avoided, enabling the visualization of vesicle bilayers and internal structures under conditions that are almost native. Hence, cryo-TEM offers better precision for biophysical and structural analysis, but it necessitates specific tools and knowledge. Comprehensive EXM characterization requires both TEM techniques. Whereas cryo-TEM is more informative for mechanistic and structural studies, particularly when assessing engineered or functionalized EXMs, conventional TEM is best for routine screening and batch quality control [50].

#### Atomic Force Microscopy (AFM)

The limitations of conventional characterization techniques are addressed by AFM, a nanotechnology tool for the investigation of a single EXM [51]. It creates three-dimensional images without the need for fixation, staining, or extremely high temperatures. EXM samples are spread out over a mica surface and allowed to air dry at room temperature in this procedure. After cleaning with ultrapure water, samples are dried with nitrogen gas. Antibody-coated mica can capture EXM with specific antigens for targeted imaging. EXM are observed using AFM with silicon or silicon nitride probes, then analyzed with specialized AFM software [50]. This technique has significantly enhanced our understanding of EXM by providing insights at single-vesicle and sub-vesicular levels, offering detailed information about structural, biophysical, and biomolecular properties of subcellular structures including DNA, membrane proteins, and vesicles [36]. AFM proves particularly effective for quantifying and analyzing EXM number, structure, biomechanics, and biomolecular content in diverse populations, including tumor samples, with capability to measure out-of-plane dimensions of nano-objects with sub-nanometer accuracy.While AFM offers valuable insights into EXM properties, certain limitations exist. Variations in sample conditions including native sample state, AFM tip condition, temperature, humidity, and probe-sample pressure can affect accuracy and reproducibility. Additionally, scan speed changes may influence measurements, potentially leading to structural and biomechanical data variations across different experimental setups [50].

#### Flow Cytometry

One molecular method for describing exosomal surface proteins is flow cytometry. Additionally, it makes it possible to measure the EXM' size and structure.Since it can identify the cellular origin of individual EVs, flow cytometry is one of the most widely used methods for EV analysis. When employing this method to isolate and characterize EXM, the initial sample amount is crucial. As a result, ultracentrifugation is still one of the most dependable methods, followed by electron microscopy, and western blotting. None of these methods, meanwhile, appear to have any potential for use in clinical or diagnostic research [36]. The latest generation of digital flow cytometry instruments, including the A50-MicroPLUS and Apogee systems, still rely on optical signal monitoring with questionable accuracy and resolution, despite lowering measurement limits to approximately 100 nm. Consequently, both magnetic carrier particles and immunological techniques must be used together for flow cytometric analysis of EXM. A practical challenge with this combination involves multiple EXM potentially binding to a single magnetic bead, reducing flow cytometry detection sensitivity [14]. Nevertheless, flow cytometry offers a reliable method for reproducible clinical sample analysis. It enables determination of EXM size and structure while examining various physical and chemical properties of cells and suspended particles. Using forward scattered light (FSC), conventional flow cytometers can quantify particles larger than 300 nm but cannot detect smaller structures, making direct EXM detection impossible with standard instruments. Flow cytometers operate by directing laser beams with specific wavelengths through fluid streams containing suspended particles. Particle presence in samples determines light scattering patterns and intensity [36].

#### Western Blot Analysis

Unlike flow cytometry, Western blot analysis does not examine intact vesicles; instead, sample preparation involves vesicle lysis and protein denaturation. Western blotting represents a commonly employed technique for EXM investigation due to its widespread availability, user-friendly nature, and ability to identify both internal and external proteins. However, major limitations include lack of multiplexing capability and variable specificity and reproducibility resulting from antibody selection [40]. In typical protocols, equivalent volumes of RIPA buffers are added to EXM preparations for lysis. Samples are standardized for protein content after BCA assay, with approximately 35 μg loaded per lane (with the exception of CSF-derived EXM isolated using ExoQuick, which are loaded at half concentration).EXM preparations undergo separation in 5–20% SDS–polyacrylamide gradient gels, electrophoretic transfer to nitrocellulose membranes, and denaturation by boiling at 99°C for 5 min in loading buffer containing β-mercaptoethanol [52]. After blocking in 5% non-fat dry milk in 1 × TBS-T (0.5% Tween-20) at room temperature, membranes are incubated with primary antibodies targeting common EXM markers, including rabbit anti-Calnexin, mouse anti-TSG101, anti-RAB11, anti-NCAM, or albumin. Following primary antibody incubation, membranes undergo washing with TBS-T (0.5% Tween-20) [36].

### Strategies for Advanced EXM Engineering

#### Exosome Surface Engineering

**Fig. 4:**
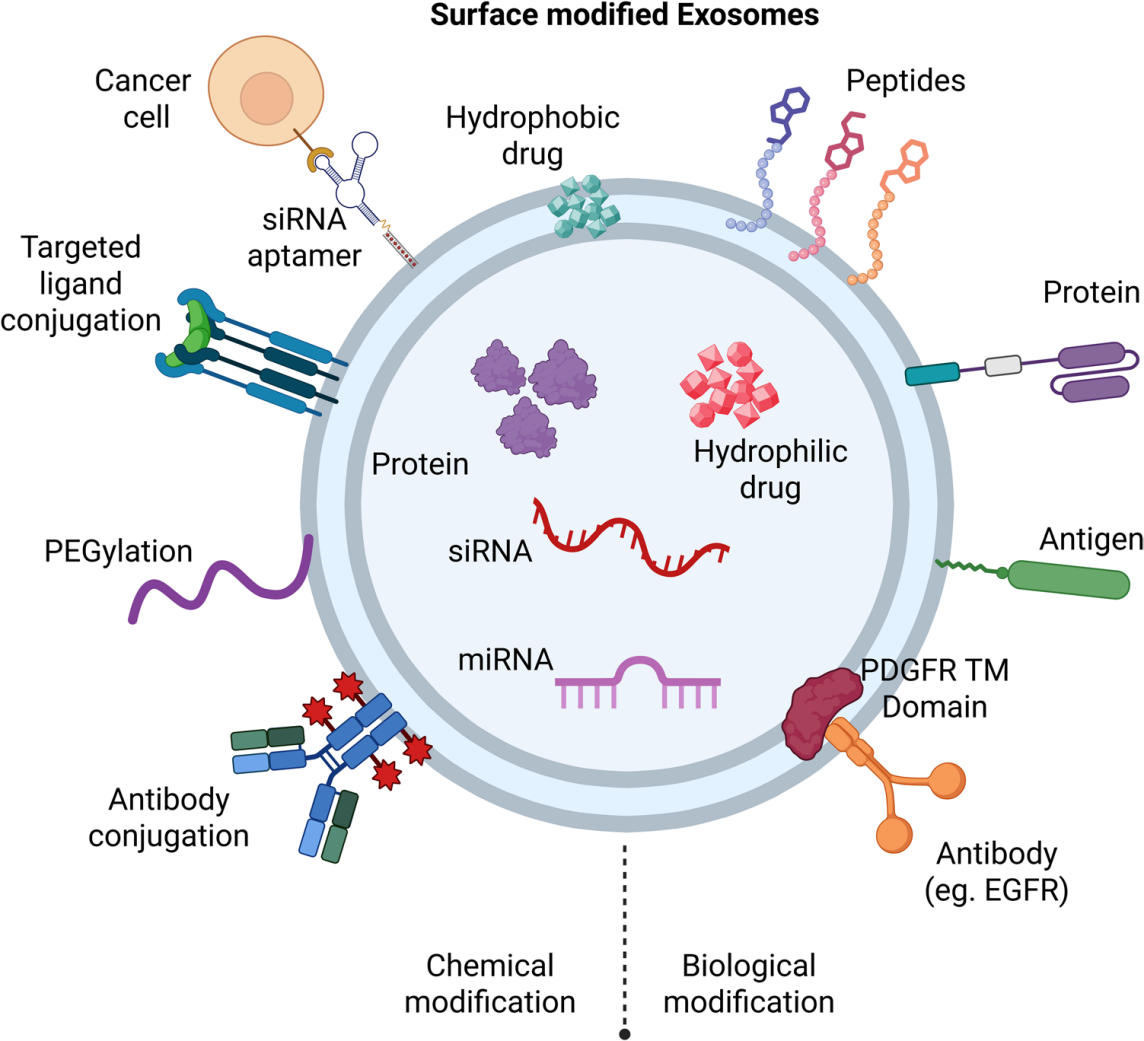
Schematic representation of Surface modification strategies for targeted delivery of Exosomes. *Different engineering methods are applied to increase the targeting ability of EXM by altering their surface. These involve genetic modification of donor cells to make them produce targeting ligands or fusion proteins on the exosomal surface, chemical conjugation of targeting moieties like antibodies, peptides, or aptamers, and click chemistry for site-specific stable ligand conjugation.

To load cargo into EXM, the membrane barrier must be bypassed. EXM functions are dependent on surface features, which engineering techniques can alter. Surface alteration is accomplished through chemical or genetic engineering. Genetic engineering combines target molecules with membrane proteins. Protein overexpression in donor cells necessitates plasmid creation. Endogenous EXM vary in composition and amount, reflecting the cell's pathophysiology [53]. Chemical changes enable the addition of molecules while preserving membrane integrity. Covalent bonds are more stable than noncovalent interactions. Click chemistry improves exosomal functioning and drug delivery. EXM can cross the blood–brain and placental barrier. This makes them effective for delivering medications to the brain, such as those used to treat Alzheimer's and Parkinson's disease [54]. Toxic substances hinder the use of covalent changes. EXM membranes can combine with liposomes during hybridization. Hybrid membranes enhance stability, circulation, and cellular absorption. EXMs have been used to transport macromolecules, which are generally defined as biological molecules like peptides, proteins, and nucleic acids that have molecular weights greater than 1,000 Daltons [55]. Shtam *et al*. discovered that trypsin treatment preferentially eliminated surface proteins from plasma EXM while preserving integrity and slightly reduced size. Mass spectrometry revealed 259 proteins, of which 79 were removed, impacting the ILK and FAK pathways. Surface plasma proteins may obscure tissue-specific features, impacting diagnosis and treatment [56]. Using extrusion, Khongkow *et al*. enhanced blood–brain barrier penetration by coating EXM with gold nanoparticles (AuNPs). This preserved targeting properties and improved brain cell interaction. The modified EXM showed superior targeting over unmodified nanoparticles [57]. Khongkow *et al*. used extrusion to coat EXM with gold nanoparticles (AuNPs), which improved blood–brain barrier (BBB) penetration while keeping targeting. This alteration increased brain cell contacts and transport, demonstrating better targeting than untreated nanoparticles. Guo *et al*. created neuron-targeting EXM (Que-mAb GAP43-Exo) that delivers quercetin to ischemic neurons with high GAP43 expression. These EXMs lowered ROS (Reactive oxygen species) via the Nrf2-HO-1 pathway, increasing neuronal survival. In a rat stroke model, they decreased infarct volume and improved recovery, indicating potential for ischemic stroke treatment [28]. Scientists are improving bio-nanoparticles like EXM for better disease detection, treatment, and drug delivery to specific cells while avoiding immune system attacks. By modifying EXM surfaces, they can more accurately target diseases like cancer and brain disorders, using proteins and peptides to guide them to the right cells**.** These engineered EXMs show promise for precise treatments, such as delivering drugs directly to tumors or ischemic brain tissues, reducing side effects, and enhancing effectiveness [58]. Ma *et al*. increased EXM using fluorinated peptide dendrimers (FPG3), which improved stability, transport, and activity. These modified EXM (exo-FPG3) increased absorption in HUVECs (Human umbilical vein endothelial cells). Fluorine alterations boosted cytosolic release, which improved EXM-based drug delivery and tissue regeneration [59]. Li *et al*. created a surface-modified contact lens (ACSM-PCL) with antibody-conjugated microchambers for detecting cancer-related EXM in tears. This lens permitted selective EXM collection and visualization while remaining transparent and strong. ACSM-PCL discovered exosomal cancer biomarkers, providing a noninvasive diagnostic tool [60]. To bypass this limitation and improve therapeutic accuracy, scientists have developed numerous surface modification methods to functionalize EXM for targeted delivery. The aim of these methods is to increase the biodistribution, cellular uptake, and tissue-specific accumulation of EXM by introducing targeting ligands, antibodies, peptides, or genetically engineered membrane proteins on the surface. Methods like donor cell genetic engineering, chemical conjugation, and click chemistry have been utilized to provide stable and functional surface modifications. The programmed EXM interact selectively with receptors that are upregulated on the diseased cells, thus reducing off-target effects and improving therapeutic potency. Figure 4 is a schematic representation of the key surface engineering approaches employed to functionalize EXM for the targeted delivery of drugs and genes.

Cargo loading EXM can be injected externally (after isolation) or internally (during creation). Cargo loading into EXMs is a vital step for therapeutic applications and may be accomplished through two basic strategies: passive and active loading. Exogenous loading involves both passive diffusions, in which cargo enters passively, and aggressive loading, which disturbs membranes. Passive loading involves incubating EXM with therapeutic molecules, which is effective for tiny (40 Da), hydrophobic substances, but has low encapsulation effectiveness [24]. It is dependent on cargo hydrophobicity and incubation period, although it is difficult to manage. Active loading procedures, such as electroporation, sonication, and extrusion, temporarily disrupt the EXM membrane to increase drug loading [61]. Active loading enhances capacity but may induce membrane damage, necessitating further purification. Endogenous loading entails altering donor cells to load EXM with cargo. Concentration, temperature, and transfection can all help enhance loading efficiency [62]. EXM can be loaded with medications either passively or actively. Passive loading is simple yet inefficient, as it relies on natural absorption. Active loading promotes absorption using methods such as electroporation, although it may harm EXM. Passive approaches are safer, whereas active methods increase loading but reduce stability [63]. EXM can be designed to transport proteins for targeted medication delivery. One way is to genetically change donor cells such that certain proteins are overproduced and spontaneously packed into EXM. However, this mechanism may alter cell equilibrium and result in unintended protein loading. Advanced sorting processes are required to increase precision and efficiency [58]. Active loading procedures, including electroporation, sonication, and extrusion, temporarily disrupt the EXM membrane to increase drug loading. Table II provides a summary of surface-engineered EXM used in targeted drug delivery, including the therapeutic agents, target diseases, modification strategies, and respective outcomes.

**Table II:** Various Surface-modified Exosome-based Drug Delivery Systems Highlighting the Therapeutic Agents and Targeted Disease Indications

Drug	Diseases	Surface modification/Targeting agent	Outcome	Ref
Doxorubicin	Cervical cancer	PEGylation, folate targeting ligand	Enhanced drug delivery and selective targeting of cancer cells	[64]
Si RNA	Lung cancer	Anti – EGFR antibody	Increased stability, specific tumor targeting, and silencing	[65]
Paclitaxel	Ovarian cancer	N- acetylglycosoamine(GlcNA) Modification	Improved anti-tumor effect and targeted delivery	[66]
Mi RNA −21	Glioblastoma	T7 peptide, lipid modifications	Targeted delivery to endothelial cells, downregulation of mi RNA −21	[67]
CRISPR -Cas9	Sickle cell disease	Transferrin receptor-targeted EXM	Targeted gene editing and reduction in off-target effects	[68]
Insulin	Type 1&Type 2 Diabetes mellitus	CD63-targeting peptide	Enhanced cellular uptake and prolonged therapeutic effect	[69]

Electroporation has been frequently employed for siRNA distribution, with encapsulation efficiency ranging from 60 to 80 percent, albeit it can cause aggregation and structural damage.

Sonication of paclitaxel into EXM yielded ~ 28% loading efficiency, providing a superior balance of effectiveness and stability compared to passive approaches. More modern approaches, such as SEAL (Sonication and Extrusion-assisted Loading), have proven up to tenfold greater encapsulation efficiency than passive loading, as well as improved repeatability. However, active loading procedures may damage EXM integrity, necessitating extra purification and optimization for clinical application [70]. Purushothaman *et al*. demonstrated that serglycin is required for protein cargo loading in tumor EXM, which affects their activity. Serglycin knockdown decreased exosomal proteins and inhibited tumor-promoting actions, indicating that it may be a cancer target [71]. Phospholipase D2 (PLD2) and the phosphatidic acid it produces are essential for EXM formation and cargo loading. PLD2 acts downstream of ARF6, affecting the formation of extracellular vesicles and their involvement in cell communication. Understanding this route may give insights into modulating EXM production for medicinal purposes [72]. EVs are attractive CRISPR/Cas9 delivery carriers because of their biocompatibility and stability. They provide a safe alternative to viral and synthetic vectors. Researchers are improving EV engineering to ensure effective freight loading and targeted delivery. These developments may improve clinical gene therapy applications [73]. Tanziela *et al*. created EXM-based carriers (D-AgNCs-ExoU87) containing DOX and AgNCs for targeted, pH-sensitive drug delivery in glioblastoma. This strategy improves tumor targeting and emphasizes EXM's promise in smart cancer treatment (75). Malhotra *et al*. employed EXM containing GAPDH to transport transferrin and lactoferrin, which increased uptake and targeted lysosomes. This work demonstrates EXM's potential for regulated drug delivery (76). Goetzl *et al*. discovered increased BACE-1 and amyloid proteins in astrocyte-derived EXM from Alzheimer's patients, tying them to amyloid pathology. This work proposes ADE cargo as biomarkers and therapeutic targets (77). Patel *et al*. emphasized EXM as nanocarriers for cardiovascular treatment, emphasizing cargo loading techniques and improved targeting. They stressed their potential in atheroprotective therapy while resolving issues with loading efficiency and stability (78). João *et al*. found LAMP2A-mediated cargo loading into EXM, like the CMA and e-Mi pathways. This method allows for the selective inclusion of proteins, which influences intercellular communication and has the potential for therapeutic applications (79). Azar *et al*. developed iPSC-derived EXM containing miRNA to improve neuronal targeting and spinal cord injury healing. These EXMs decreased inflammation, increased neuron uptake, and aided functional recovery, demonstrating their therapeutic potential [73]. Table III consolidates several EXM-based delivery methods with indications of therapeutic cargo type, target disease, encapsulation efficiency, release profiles, and stability characteristics.

**Table III:** Summary of Exosome-based Delivery Strategies, Detailing Therapeutic Cargo Types, and Target Diseases

Methods	Cargo	Diseases	Encapsulation efficiency	Release Profile	Stability	Reference
Passive loading	Doxorubicin	Cervical cancer	~ 10%	Slow, sustained over 72 h	High (membrane intact)	[74]
Electroporation	siRNA	Lung Cancer	Up to 60–80%	Initial burst, then sustained	Risk of aggregation, needs cleanup	[75]
Sonication	Paclitaxel	Ovarian cancer	~ 28%	Moderate, quicker than passive	Moderate, potential structural impact	[76]
Extrusion	Curcumin	Ulcerative colitis	~ 30–40%	Gradual release, less burst effect	Slight alteration in morphology	[77]
SEAL(Sonication + Extrusion)	Doxorubicin	Cervical/Breast cancer	~ 70–80%	More uniform, sustained release	Improved reproducibility	[78]

#### Genetic Engineering

**Fig. 5:**
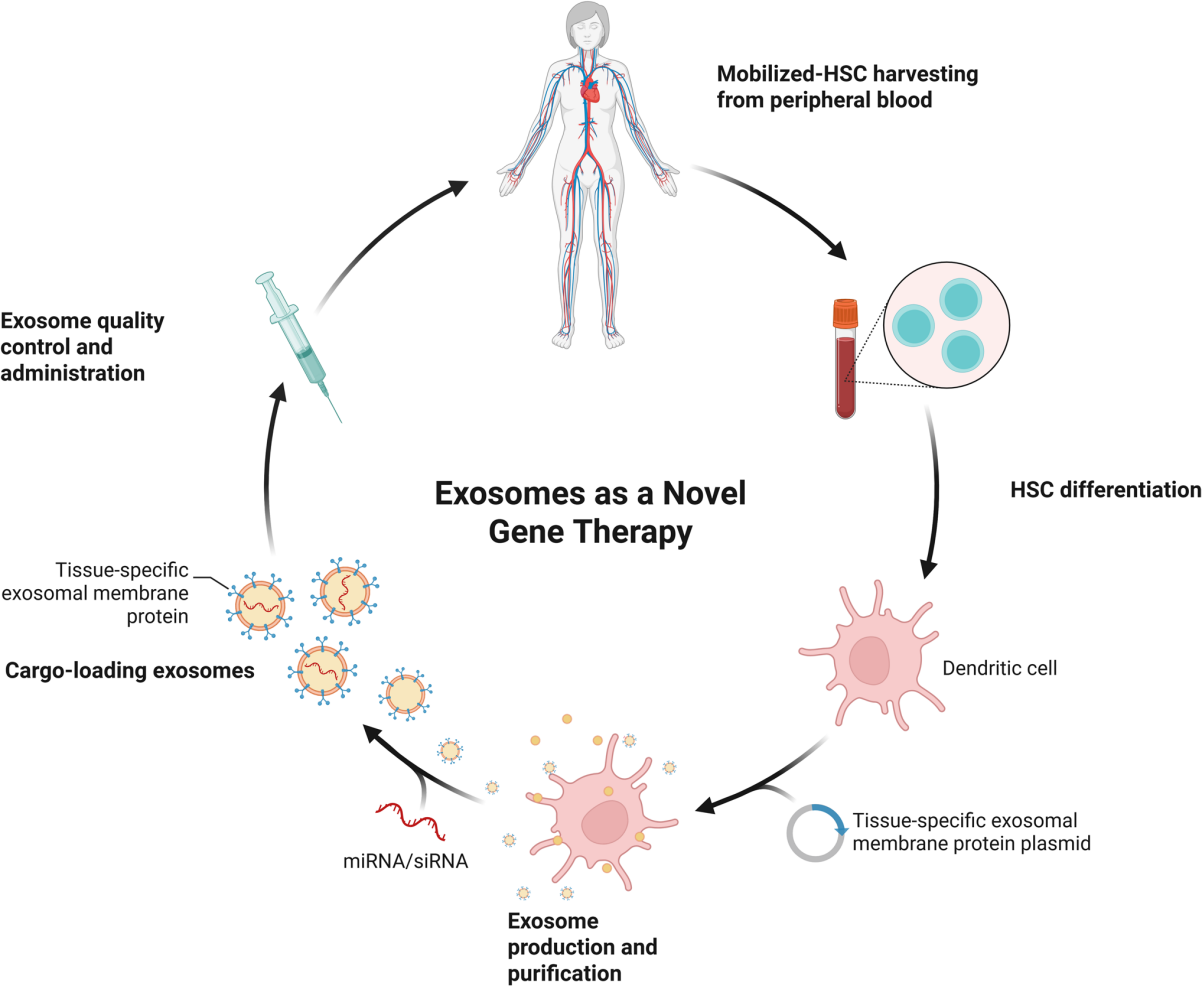
Exosome-mediated gene therapy using autologous stem cell-derived systems *Depicts the mechanism of producing self-derived EXM for gene therapy, from harvesting HSC and cell differentiation to engineering EXM, loading cargo, and administration.

Recent developments in EXM engineering have allowed for the utilization of autologous, cell-derived EXM as safe and efficient delivery carriers of gene therapy with reduced immunogenic risks and improved targeting efficiency. Hematopoietic stem cells (HSCs) are initially mobilized and collected from the peripheral blood of the patient. The cells are later differentiated into dendritic cells. EXM are generated and purified from the engineered cells, which have been transfected with a tissue-specific exosomal membrane protein plasmid. Therapeutic nucleic acids, like miRNA or siRNA, are then loaded into the EXM, and membrane proteins are expressed to be delivered in a targeted manner. The final EXM preparation is then quality checked and infused back into the patient, facilitating autologous, targeted gene therapy with little immunogenicity (Fig. 5). Zheng Ren *et al*. discovered that MSC-derived EXM containing miR-146a/miR-155 decreased IFNγ expression and had anti-inflammatory benefits in arthritis. Their findings point to EXM as a possible cell-free treatment for immunological regulation (84). To improve immunological activation, Morishita *et al*. created CpG-SAV-exo, a modified EXM that co-delivers CpG DNA and tumor antigens to dendritic cells. These EXM enhanced antigen presentation in mice with cancer and demonstrated more potent antitumor effects (85). Youngue *et al*. created neprilysin-enriched RVG-displaying EXM that target α7-nAChR and break down β-amyloid to cure Alzheimer's disease. These EXM specifically targeted the hippocampus in mice and were more effective than stem cell-derived EXM at lowering Aβ40 levels. Additionally, they demonstrated therapeutic promise by increasing IL10 expression and decreasing inflammation (88). Li *et al*. created a hydrogel based on EXM to stimulate macrophages to treat ovarian cancer. It improved antigen presentation and tumor phagocytosis with X-ray radiation and an inhibitor. Additionally, it showed promise in the treatment of triple-negative breast cancer [73]. To transfer CRISPR/Cas9 plasmids to chondrocytes in osteoarthritis, Liang *et al*. created a hybrid EXM. The genetic alteration decreased cartilage degradation by targeting MMP-13. This method demonstrates how genetic engineering may be used to cure osteoarthritis [73]. Sarkar *et al*. created CAP-EXM by combining AAV capsid peptides with Lamp2b for targeted CNS medication delivery. These EXM efficiently conveyed GFP genes, with a 20-fold increase in transfer over control EXM. This strategy has the potential for CNS therapeutics [79]. Sarkar *et al*. created genetically modified EXM (GEMINI-Exos) that included antibodies targeting T-cell CD3 and EGFR, as well as immune modulators such as PD-1 and OX40L, to activate T cells and target triple-negative breast cancer cells. This approach unearths the potential of EXM-based immunotherapy for effective anti-cancer immunity [80]. Wei *et al*. designed genetically engineered SNHG12-loaded cerium-macrophage EXM (Ce-Exo) as targeted therapeutics for atherosclerosis treatment. *In vivo* studies showed that Ce-Exo was superior at targeting and clustering plaques, modulating the inflammatory environment, and repairing endothelial cell damage. This method highlights the potency of EXM-based therapeutics in atherosclerosis treatment and enhancing biomedicine [81]. Mesenchymal stem cells (MSCs) possess therapeutic potential for COVID-19 by modulating the immune system and cytokine storm through paracrine actions. Genetic engineering, surface modification of cells, and applications of nanotechnology are all highlighted as being crucial to optimizing MSC-based treatments. All these aim at enhancing the therapeutic efficacy of MSCs against COVID-19 [82]. Lin *et al*. used genetic modification to modify Huc-MSC-derived EXM with targeting peptide HSTP1, making them selectively bind to activated hepatic stellate cells (aHSCs). The method enhances EXM targeting and therapeutic efficiency for liver fibrosis treatment. The system provides an accurate method of targeting aHSCs in complex liver tissues [83].

#### Applications

The clinical potential of EXM in ischemic stroke was explored by Li *et al*. who pointed toward the advantage of repair effects and the capacity for cell communication. EXM derived from stem cells and other origins possesses protective activity with enhanced EXM with modified efficiency [84]. EXM are now being explored in the clinical environment in the context of liquid biopsies, therapeutic treatment, and drug delivery. They have benefits over standard biopsies and can act as non-invasive biomarkers for a variety of disorders, including cancer, diabetes, and cardiovascular disease. EXM may also be designed for targeted treatment and drug administration, which improves stability and bioavailability. They have shown promise in treating illnesses such as stroke, COVID-19, and neurological disorders [85]. EXM is used for targeted drug delivery, gene therapy, and protein delivery, crossing barriers like the blood–brain barrier. They carry therapeutic agents for diseases like cancer and neurodegenerative disorders. EXM-based therapies offer non-invasive, specific treatments [14]. EXM (30–150 nm) are used in liquid biopsy to analyze biofluids such as tumor cells, nucleic acids, and proteins. They are used to diagnose cancer, pregnancy issues, cardiovascular ailments, and organ transplantation, providing diagnostic benefits [86]. EXMs are utilized to diagnose and treat pancreatic disorders by serving as indicators for aberrant cellular function. They play a role in the diagnosis and treatment of cancer and inflammatory pancreatic disease, and they might be targeted in future therapeutic strategies [87].

EXM targets colorectal cancer (CRC) growth, drug resistance, and immune responses. They are diagnostic biomarkers, drug delivery vectors, and possible vaccine vehicles. More studies are needed to establish their therapeutic applications in CRC diagnosis and therapy [88]. EXM is a potential diagnostic biomarker and anticancer therapeutic agent, particularly in head and neck squamous cell carcinoma (HNSCC) monitoring and therapy. However, further research is required for practical use [89]. Lung cancer cell-derived EXM (LCCDEs) are now gaining focus in their functions in lung cancer growth, diagnosis, drug development, and prognosis (68). They have promise as biomarkers and effective drug delivery vectors because of their natural potential to carry biomolecules, low toxicity, and ability to cross biological barriers [90]. EXM, with their ability to carry mRNAs, miRNAs, proteins, and antigens, are key regulators of the immune system and are becoming novel vaccine development and delivery vehicles with potential applications in cancer immunotherapy and infectious disease control [91]. EXM play an important role in tuberculosis (TB) by influencing immunological responses such as inflammation and antigen presentation, and they have potential as a new delivery method for anti-TB vaccines and therapeutics [92]. EXM are being investigated as a promising platform for cancer vaccine development, with examples including the prostate cancer vaccine Provenge® (sipuleucel-T), which uses dendritic cells in cancer immunotherapy, and ongoing research into engineered EXM to improve vaccine efficacy and immune system priming [93]. Surface-engineered EXM derived from dendritic cells and loaded with respiratory syncytial virus (RSV)-specific peptides shows potential as a vaccine platform, demonstrating the ability to stimulate virus-specific immune responses. However, further development is required to optimize their capacity to prime CD8 + T cells effectively [94]. An immunoinformatics approach has been used to design multi-epitope vaccines against Mycobacterium tuberculosis by selecting immunogenic EXM proteins, incorporating antigenic B-cell, helper T-lymphocyte, and cytotoxic T-lymphocyte epitopes, and using the TLR4 agonist RpfE as an adjuvant. This approach aims to develop globally effective TB vaccines with minimal adverse effects [95].

#### Challenges and Limitations

One of the most significant problems in developing EXM (EXM)-based therapies is the poor yield of natural EXM, which significantly restricts their availability for large-scale clinical applications. Current techniques of isolating EXM from cell culture supernatants or bodily fluids generate limited numbers and frequently exhibit batch-to-batch variability due to changes in donor cell conditions, culture medium, and separation methodologies [96]. While genetic and biochemical engineering can improve EXM output and consistency, scalable manufacturing that meets clinical requirements remains challenging. EXM-based medicines also face major regulatory and quality control problems due to their complex composition and biological origin. Regulatory organizations such as the FDA and EMA demand extensive characterization of EXM products, including their size, cargo composition, source, mode of action (MOA), and repeatability across production batches. EXMs frequently exhibit variability in their RNA, protein, and lipid profiles, hindering the creation of repeatable potency tests and quality benchmarks required by Good Manufacturing Practices (GMP)[97]. Another significant hurdle is the difficulty in long-term storage and stability of EXM. EXM are vulnerable to environmental changes and can degrade or lose bioactivity if not kept at ultra-low temperatures (usually −80°C), which is expensive and logistically hard. Short-term storage at 4 degrees Celsius is inadequate to retain their structural integrity and functional potency, and there is currently no consensus on effective cryoprotectants or preservation techniques [98]. In terms of clinical pharmacokinetics, EXM has quick clearance, a short half-life in circulation, and nonspecific biodistribution following systemic injection. Surface engineering or integrating targeting ligands may increase tissue specificity and therapeutic effectiveness, but these techniques involve extra validation and regulatory obstacles. The capacity of EXM to overcome biological barriers, such as the blood–brain barrier (BBB), is uneven and represents a significant constraint in the treatment of neurological illnesses [99]. Furthermore, difficulties like as off-target absorption, poor drug loading, unpredictable release kinetics, and short circulation duration continue to undermine the clinical efficacy of EXM-based delivery systems [100]. From a clinical translation standpoint, ethical and safety concerns must be addressed. EXM produced from human cells may transmit pathogenic pathogens or carry oncogenic material, particularly if not adequately screened or treated. There are further issues with EXM treatments provided in uncontrolled environments with poorly described materials, generating concerns about patient safety and consent. Clinical approval and acceptance are further delayed due to a lack of unified worldwide regulatory criteria. Finally, high manufacturing costs, complicated storage requirements, and the need for uniform methods across isolation, purification, and engineering processes have yet to be answered. Overcoming these technological, regulatory, and ethical challenges is critical to effectively turning engineered EXM into safe, efficacious, and widely available therapeutic medicines [98].

#### Future Perspectives

Current trends in EX engineering center on optimizing their performance through surface modification to enhance the specificity of targeting, stability, and capacity for cargo loading. EXM was designed with transferrin receptors to target cancer cells that produce high quantities of this receptor, boosting delivery accuracy. EXM can transport therapeutic biomolecules (RNA and DNA) thanks to genetic alterations such as CRISPR/Cas9, making them possible vectors for gene therapies or mRNA vaccinations, providing innovative treatments for genetic illnesses and malignancies. The role of artificial intelligence (AI) is to optimize EXM engineering by accessing data to forecast the optimum alterations for targeting and cargo delivery. For example, AI systems can detect surface indicators that boost EXM effectiveness in cancer therapy. AI may also assist in optimizing EXM manufacturing by assessing circumstances to enhance yield and consistency, which is critical for clinical applications. Furthermore, AI might broaden EXM utilization by discovering novel therapeutic targets and tailored medical applications, such as RNA delivery for specific disorders.

## Conclusion

EXMs are an exciting new frontier in targeted therapeutics due to their distinctive nanoscale attributes and capacity to transport biomolecules such as proteins, nucleic acids, and small molecules. While the clinical use of EXM is hindered by obstacles such as quick immune clearance, inefficiencies in cargo loading, and targeting constraints, recent improvements in EXM engineering have greatly improved their attractiveness as therapeutic delivery vehicles. Approaches like genetic engineering, surface functionalization with ligand targeting, and synthetic vesicle construction have been promising to surmount these barriers. Furthermore, engineered EXM has exhibited potential in various therapy areas, ranging from cancer treatment to gene therapy and regenerative medicine, most notably in surmounting barriers like the blood–brain barrier. Manufacturing-related problems, quality controls, and regulatory modalities remain strong barriers to clinical translation. As technology advances, developing trends such as multifunctional EXM and AI-driven optimization promise to further optimize and maximize the therapeutic application of EXMs. EXM engineering is poised to transform precision medicine delivery, unlocking new avenues for customized healthcare solutions.

## Data Availability

Data will be provided on request.
